# Associations Of Childhood Socioeconomic Position, Affective Problems, And Later-Life Cognition

**DOI:** 10.1093/geroni/igaf122.109

**Published:** 2025-12-31

**Authors:** Anja Leist, Sarah-Naomi James, Yiwen Liu, Marcus Richards, Anouk Geraets

**Affiliations:** University of Luxembourg, Esch-sur-Alzette, Grevenmacher, Luxembourg; University College London, London, England, United Kingdom; University College London, London, England, United Kingdom; University College London, London, England, United Kingdom; University of Luxembourg, Esch-sur-Alzette, Grevenmacher, Luxembourg

## Abstract

Socioeconomic inequalities may act on affective and cognitive health from early life through later-life. This study tested to what extent the association of childhood socioeconomic position (SEP) with later-life cognitive ageing is explained by life-course accumulative affective problems. Data were from the MRC National Survey of Health and Development (n = 1,593; 52.6% women). Cognitive ageing was assessed through a battery of neuropsychological tests. Later-life cognitive function was assessed at age 69, and cognitive decline was measured across ages 53, 60–64 and 69. Childhood SEP was derived from overcrowding, essential household amenities, housing condition, and paternal occupation. Affective problems measured at ages 13–64 were categorized into case-level thresholds (never/once/twice or more). Multiple linear and logistic regression analyses and structural equation modeling tested associations between childhood SEP, affective problems, and later-life cognitive ageing. Low childhood SEP was associated with lower scores on later-life (age 69) verbal memory (B=-1.87[-2.48;-1.25]), letter search speed (B=-9.98[-17.57;-2.40]), letter search accuracy (B=-0.90[-1.44;-0.37]), cognitive state (B=-2.14[-2.82;-1.46]), and accelerated decline (between ages 53-69) in letter search accuracy (B = 0.82[0.17;1.46]), but slower decline in verbal memory (B=-0.27[-0.51;-0.00]) compared to high childhood SEP. Low childhood SEP was associated with increased risk of affective problems once (OR = 1.37[1.09;1.73]) and twice or more (OR = 1.32[1.01;1.71]). The association between childhood SEP and later-life letter search accuracy was partly explained (6.3%) by incidental affective problems. Although improving affective health across the life course may enhance later-life cognitive ageing, it could only marginally explain the association between childhood SEP and later-life cognitive ageing.

